# ERPLAB: an open-source toolbox for the analysis of event-related potentials

**DOI:** 10.3389/fnhum.2014.00213

**Published:** 2014-04-14

**Authors:** Javier Lopez-Calderon, Steven J. Luck

**Affiliations:** Department of Psychology, Center for Mind and Brain, University of California-DavisDavis, CA, USA

**Keywords:** event-related potential (ERP), matlab toolbox, open source, data analysis, signal processing

## Abstract

ERPLAB toolbox is a freely available, open-source toolbox for processing and analyzing event-related potential (ERP) data in the MATLAB environment. ERPLAB is closely integrated with EEGLAB, a popular open-source toolbox that provides many EEG preprocessing steps and an excellent user interface design. ERPLAB adds to EEGLAB’s EEG processing functions, providing additional tools for filtering, artifact detection, re-referencing, and sorting of events, among others. ERPLAB also provides robust tools for averaging EEG segments together to create averaged ERPs, for creating difference waves and other recombinations of ERP waveforms through algebraic expressions, for filtering and re-referencing the averaged ERPs, for plotting ERP waveforms and scalp maps, and for quantifying several types of amplitudes and latencies. ERPLAB’s tools can be accessed either from an easy-to-learn graphical user interface or from MATLAB scripts, and a command history function makes it easy for users with no programming experience to write scripts. Consequently, ERPLAB provides both ease of use and virtually unlimited power and flexibility, making it appropriate for the analysis of both simple and complex ERP experiments. Several forms of documentation are available, including a detailed user’s guide, a step-by-step tutorial, a scripting guide, and a set of video-based demonstrations.

## INTRODUCTION

The event-related potential (ERP) technique is widely used in basic and translational research on sensory, cognitive, affective, and motor processes (Hillyard and Picton, 1987; Rugg and Coles, 1995; Kutas and Dale, 1997; Luck, 2005, 2012b; Luck and Kappenman, 2012). ERPs provide a non-invasive means of measuring brain activity in humans, and its millisecond temporal resolution and coarse spatial resolution complement the coarse temporal resolution and fine spatial resolution of functional magnetic resonance imaging (fMRI). In addition, ERPs are relatively inexpensive to record and well tolerated by subjects who cannot easily participate in fMRI studies.

The last decade has seen an explosion in the development of commercial systems for recording the electroencephalogram (EEG), including inexpensive systems of good quality and more expensive systems with greatly improved features and performance (e.g., driven right leg circuits, 24- or 32-bit resolution, ultra-high input impedance). This has led to a dramatic increase in the number of installed EEG/ERP systems. However, a significant impediment to the optimal use of these systems has been the lack of high quality, full featured, and widely available ERP analysis tools. Several commercial packages are available, but they are very expensive, lacking in important features, difficult to customize, and inconvenient to use for state-of-the-art research. In addition, commercial analysis packages necessarily focus on methods that have been used in prior research and are widely known, whereas science requires the constant creation of new analysis methods. Researchers must therefore have access to software that easily allows the creation and dissemination of new analysis techniques.

We have therefore created an open-source ERP analysis package called ERPLAB toolbox that is designed to meet the needs of a wide variety of researchers. ERPLAB can be downloaded for free at http://erpinfo.org/erplab and detailed documentation can also be found at that site. ERPLAB is compatible with the EEG file formats of all major data acquisition systems, and it can import averaged ERP waveforms from other ERP analysis systems either directly or through a common text-based interchange format. ERPLAB runs on all major computer operating systems. It is designed to provide a convenient workflow, a fast learning curve, ease of use for inexperienced researchers, and virtually limitless power and flexibility for experienced researchers. The purpose of this Technology Report is to provide an overview of this package, including the underlying design principles and the core features.

## GENERAL DESIGN

### THE MATLAB PROGRAMMING ENVIRONMENT

ERPLAB toolbox operates in the MATLAB programming environment. MATLAB (The Mathworks, Inc., Natick, MA, USA) is a full-featured programming language that is widely used in science and engineering. It has several features that make it easy for novices to write small programs (scripts) and for more sophisticated users to create new data processing functions. Thousands of science-oriented mathematical functions are available in add-on toolboxes. Many of these toolboxes are available for a modest fee from The Mathworks, and many others (such as ERPLAB) are provided at no cost by individual scientists and engineers. MATLAB runs on all major operating systems, so ERPLAB can be used in a variety of computing environments. MATLAB has become the most common programming environment for cognitive neuroscientists, in part because of the widespread use of the SPM package in neuroimaging (Friston et al., 2006). MATLAB is also widely used for stimulus presentation, in part because of the availability of the Psychophysics Toolbox package (Brainard, 1997) and the Cogent Graphics package^1^. MATLAB can also be used for statistical analyses, either via the Statistics Toolbox or via communication with the R statistical environment. It is therefore broadly valuable for researchers to learn MATLAB.

The most significant disadvantages of MATLAB are that it is not free, that it is slow for some kinds of processing operations, and that it sometimes uses memory inefficiently. However, it is an order of magnitude less expensive than commercial EEG/ERP analysis systems, is extremely fast for matrix operations, and is now available in 64-bit versions that can address very large amounts of memory.

### INTEGRATION WITH EEGLAB TOOLBOX

**FIGURE 1:**
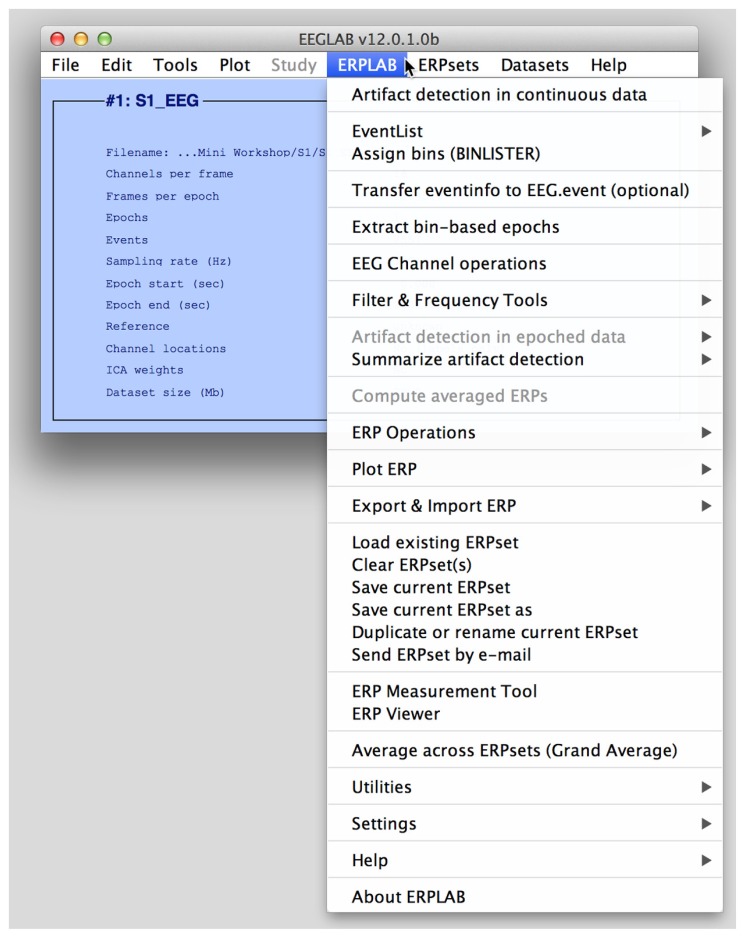
**The main EEGLAB graphical user interface, with the ERPLAB menu activated.** The Datasets menu shows a list of currently active EEG sets, and the ERPsets menu shows a list of currently active ERP sets.

ERPLAB toolbox is tightly integrated with EEGLAB Toolbox (Delorme and Makeig, 2004), a widely used MATLAB toolbox for processing the EEG. As shown in **Figure 1**, the EEGLAB graphical user interface (GUI) contains an ERPLAB menu (when ERPLAB has been installed). EEGLAB has built-in facilities for the addition of plug-ins like ERPLAB, making it possible for ERPLAB to be added onto EEGLAB in a modular fashion.

ERPLAB relies heavily on EEGLAB’s functions for: (a) importing EEG data from all major EEG data collection systems; (b) plotting EEG waveforms and EEG/ERP scalp maps; and (c) performing independent component analysis (ICA; Makeig and Onton, 2012), especially in the context of artifact correction (Jung et al., 2000a,b). ERPLAB adds several EEG processing tools to EEGLAB, including additional artifact detection functions, additional filters, a tool for recombining channels using algebraic expressions (e.g., for re-referencing), and a powerful method for categorizing event codes. These functions can be used both by researchers who are interested in the ERP processing abilities of ERPLAB and by researchers who are mainly interested in using EEGLAB to perform EEG analyses (e.g., time-frequency analyses).

EEGLAB has some built-in routines for calculating conventional averaged ERPs, but it does not emphasize ERP processing and therefore does not include many of the standard processing tools needed for ERP research. ERPLAB was created to provide these tools.

### GUI, HISTORY, AND SCRIPTING

**FIGURE 2:**
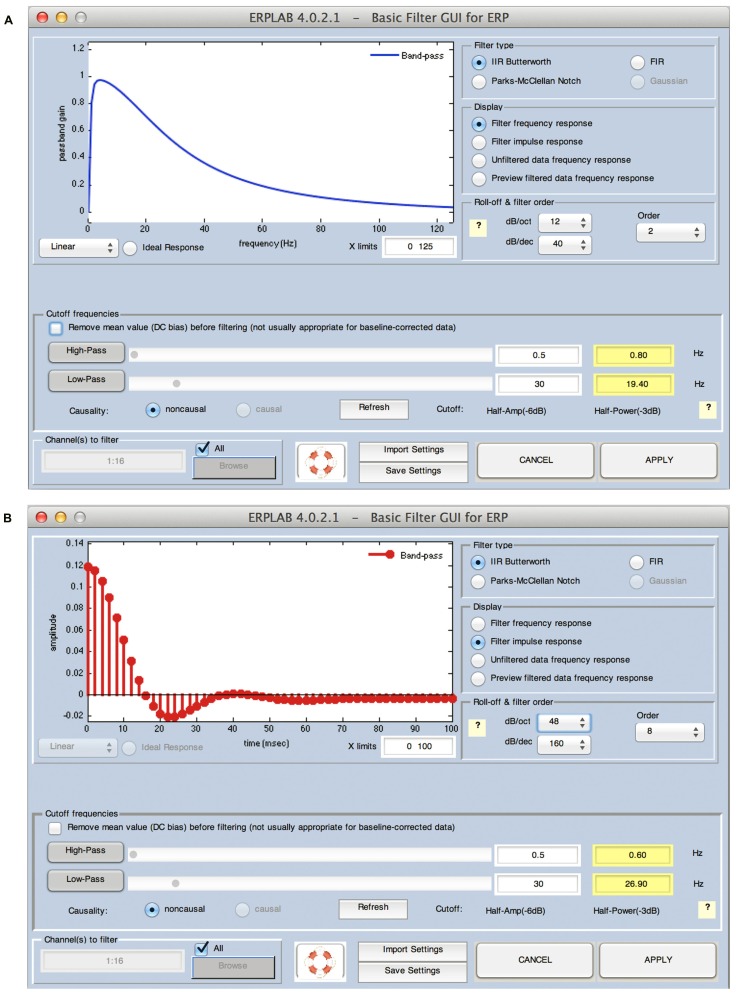
**ERPLAB’s filtering interface, which can show the frequency response function (A) or the impulse response function (B) of the currently specified filter**.

A convenient GUI is an important aspect of an ERP analysis package. It dramatically reduces the time required for both experienced and novice researchers to learn to use the package. It also makes the various options very salient, because a user can see what options are available without consulting the documentation. Consider, for example, the GUI for the ERPLAB filtering function (**Figure 2**). It has an option labeled “Remove mean value (DC offset) before filtering (not usually appropriate for baseline-corrected data).” The presence of this option in the GUI makes it obvious that this option exists, and the label of the option is fairly self-explanatory, obviating the need for the user to consult the documentation. In addition, the label indicates that this option is not usually appropriate for baseline-corrected data, thereby providing advice to novice users about when this option should be used. A well-designed GUI serves as a means of teaching users optimal practices for data analysis, and ERPLAB is intentionally designed to be an implicit teaching tool as well as an analysis tool.

Although GUIs are very useful, they can be cumbersome for processing large data sets. Imagine, for example, that a manuscript is submitted to a journal describing an experiment with 50 subjects, and a reviewer asks for a reanalysis of the data that requires changing one of the first steps in the data processing pipeline (e.g., the cutoff of a high-pass filter that was applied to the continuous EEG prior to any other processing steps). Reanalyzing the data by means of a GUI might require 2 h of pointing and clicking per subject, for a total of 100 h of effort. It is therefore very useful to be able to create automated scripts for data processing (but with the possibility of using different settings for different subjects, e.g., to deal with a broken electrode in one of the subjects). However, scripting languages are often difficult to learn, especially for researchers who do not have a computer programming background. Some commercial ERP analysis packages have automation/scripting abilities, but they are either difficult to learn or not sufficiently flexible.

One of the greatest strengths of EEGLAB is that it provides an easy-to-follow path from using the GUI to writing automated but flexible scripts. In EEGLAB, any operation that is performed in the GUI (e.g., loading a set of EEG data from the hard drive into memory) has an equivalent script command [e.g., *EEG = pop_loadset*(*‘filename’,‘S1_EEG.set’*)] that is automatically saved in a *command history*. Thus, a user can process a subject’s data using the user-friendly GUI and then use the history as the basis for a script that can be used to automatically process other subjects’ data. This approach to script-writing can be easily mastered by graduate students or postdocs who have no prior programming experience. Once this has been mastered, many users can learn to create more sophisticated scripts that implement new processing techniques. This is facilitated by the huge array of existing processing functions that are available for MATLAB, which allow users to develop new processing techniques with a relatively small number of lines of code and without advanced knowledge of mathematics.

ERPLAB uses this same approach, in which each operation that is performed in the GUI is saved in a history as an equivalent script command. We have extended this slightly, adding commands to the history whether they were called from a script or from the GUI. Because the history is stored in the same data structure as the EEG or ERP data, this provides a means of remembering the sequence of steps that was used to process a given file (e.g., when writing a manuscript two years after the data were processed). In addition, we have written an *ERPLAB Scripting Guide* that is designed to help people with no programming background learn how to write EEGLAB/ERPLAB scripts.

### DATASETS AND ERPSETS

In EEGLAB, a *dataset* is a set of EEG data and associated information from a single subject. In most commercial systems, this would correspond to an EEG file. However, a dataset can be stored in memory instead of, or in addition to, being stored in a file. Each data processing operation (e.g., filtering, re-referencing, epoching) operates on the *current dataset* and creates a new dataset, which then becomes the current dataset in EEGLAB’s GUI. Each dataset in memory appears in a *Datasets* menu (see **Figure 1**). Ordinarily, each new dataset created by applying a processing operation (e.g., filtering) is stored in memory and not saved in a file, and only the first and last datasets in a processing pipeline are saved as files. This makes it easy for the user to back up and repeat an operation (by selecting a previous dataset from the Datasets menu), without clogging the hard drive with large numbers of files.

ERPLAB inherits this scheme and adds to it by creating *ERPsets*, which store averaged ERP waveforms. A single ERPset can contain all the ERP waveforms from all the stimuli and experimental conditions for a given subject in an experiment. Each ERPset can be stored in a file or in memory, and an *ERPsets* menu provides a list of all ERPsets that are currently available in memory. Each data processing operation (e.g., filtering, making difference waves, making grand averages) operates on the current ERPset and creates a new ERPset, which then becomes the current ERPset. In practice, this scheme is very convenient for the user.

## KEY FEATURES

### PROCESSING EVENT CODES

In ERP experiments, a signal is sent from the stimulus presentation computer to the EEG acquisition computer whenever a stimulus or response occurs. In EEGLAB and ERPLAB, these signals are called *event codes* (they are known in other systems as *marker codes*, *trigger codes*, *stimulus codes*, etc.). Event codes play a central role in ERP research, because ERPs are isolated from the overall EEG by extracting segments of EEG data that are time-locked to the event codes and averaging the segments from multiple trials. Events are so central in ERP experiments that they are a part of the name of the technique (the ERP technique). ERPLAB contains several features that are designed to give users easy access to the event codes and to perform a variety of operations with them. ERPLAB does not make an intrinsic distinction between stimuli, responses, or other kinds of events: the user is given total freedom to use event codes in any way that is appropriate for a given experiment.

ERPLAB takes the event codes that are present in EEGLAB’s data and creates a special data structure called an *EVENTLIST*. This structure provides many pieces of information for each event, including a numeric code, a text label, a time of occurrence, an enable/disable flag (to mark events that should be excluded because of errors during data collection), a set of artifact flags (to indicate that specific artifacts were associated with a given artifact), and a set of user-defined flags (which can be used for many purposes, such as indicating the experimental condition in which a given event occurred).

The EVENTLIST structure can be exported as a text file, allowing it to be easily viewed with the MATLAB text editor. It can also be edited and then imported back into the EEG data. This provides a very easy way for the user to add, delete, or modify information in the EVENTLIST. For example, if an eye tracker is used concurrently with the EEG recordings, information about saccade onsets in the eye tracker’s data file can be integrated into the EVENTLIST text file and then imported back into the EEG data. Similarly, if the stimulus presentation program’s data file contains events that were not sent as event codes during the experiment, these events can be integrated into the EVENTLIST text file and then imported back into the EEG data. These events could therefore be used as the time-locking events for averaged ERP waveforms. Note that an *EYE-EEG* plug-in for EEGLAB is also available for inserting event codes for eye movements directly into the EEG data (Dimigen et al., 2011).

ERPLAB also contains tools for inserting event codes when specific features are identified in the EEG data. For example, it would be possible to automatically insert an event code at the onset of an alpha burst, an eyeblink, or a burst of muscle activity. Again, these events could be used as the time-locking events for averaged ERP waveforms.

ERPLAB also contains a sophisticated tool for determining which event codes should be averaged together. In an oddball experiment, for example, it is necessary to separately average the standard and oddball stimuli. Separate averages are computed for each electrode site, but based on the same set of events. We refer to the averaged data from each electrode site for a given set of events as a *bin* (e.g., a simple oddball experiment would have one bin for the standards and one bin for the oddballs). In most ERP analysis systems, there is a one-to-one relationship between event codes and bins, but many experiments require a more complex relationship. In a properly counterbalanced oddball experiment, for example, the letter X might be rare and the letter Y might be frequent in some trial blocks, whereas Y might be rare and X might be frequent in other trial blocks. It is therefore useful to be able to lump together all the oddball stimuli into one bin (i.e., X when X is the rare stimulus and Y when Y is the rare stimulus) and all the standard stimuli into another bin (i.e., X when X is the frequent stimulus and Y when Y is the frequent stimulus). Alternatively, it can be useful to subdivide different trials that have the same event code. For example, it can be useful to have separate bins for oddballs preceded by oddballs and oddballs preceded by standards, and it can be useful to have separate bins for oddballs followed by correct responses and oddballs followed by correct responses.

To address this fundamental need of ERP experiments; ERPLAB contains a *BINLISTER* function that provides a powerful mechanism for sorting event codes into user-defined bins. The user creates a text file with relatively abstract descriptions of the sequence of events that defines a given bin, and BINLISTER finds all sequences that match this description. For example, the following would be a description of a bin in which either of two oddball event codes (21 and 22) is preceded by either of two standard event codes (11 or 12) and followed by a correct response (event code 101) that occurred between 200 and 1000 ms after the oddball:

{11;12}.{21;22}{t<200–1000>101}

BINLISTER is both easy to use for simple experiments and capable of complex event sorting for more sophisticated experiments. If BINLISTER is insufficient for a given experiment, a sophisticated user can write a MATLAB script (or Excel macro) that sorts the events into bins, according to the special needs of that experiment. This information can then be imported back into the data using ERPLAB’s tools.

BINLISTER can also be used for analyses of behavioral data, which can then be linked with the ERP data. For example, the following bin descriptor will extract the reaction time for event code 101:

{11;12}.{21;22}{101:rt<"RT_Correct_Re⁡sponse">}

### ARTIFACT DETECTION, REJECTION, AND CORRECTION

**FIGURE 3:**
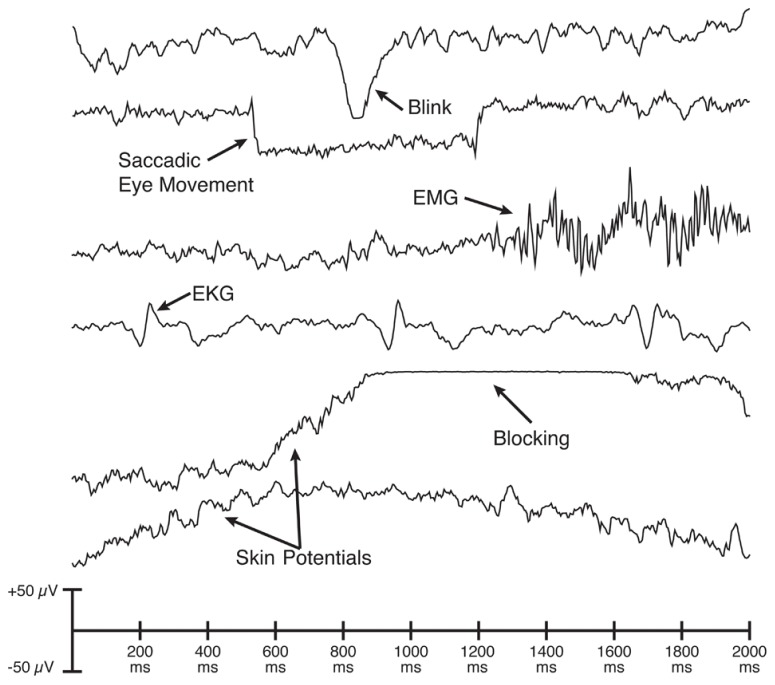
**Examples of single-trial waveforms showing common artifacts.** Each artifact has a distinctive set of properties and can therefore be detected most accurately by algorithms that are tailored to these properties.

There are many different types of artifacts that may contaminate EEG data, adding noise or confounding comparisons between conditions (see **Figure 3**). The most common and problematic artifacts are typically eyeblinks and saccadic eye movements, which derive from the strong corneal-retinal potential inside each eye (for an excellent review, see Plochl et al., 2012). Other common artifacts include muscle activity, sudden shifts in potential caused by movements, gradual shifts in voltage caused by skin potentials, the electrocardiogram, and blocking (saturation) of the amplifier or analog-to-digital converter. As illustrated in **Figure 3**, each kind of artifact has a distinctive waveform. Some artifacts also have distinct scalp distributions.

When an artifact has a stable scalp distribution, it is usually possible to use ICA to decompose the EEG data into a set of underlying components and then reconstruct the data without the component corresponding to the artifact. This effectively eliminates the artifact from the EEG. This method of *artifact correction* has become very popular for eliminating blink artifacts, which have a very stable scalp distribution and are so large that they usually appear as a single ICA component. ICA-based artifact correction is very well implemented in EEGLAB, and ERPLAB inherits this ability from EEGLAB.

However, ICA-based artifact correction has some important limitations. First, ICA cannot work for artifacts that do not have a consistent scalp distribution for a given subject (e.g., skin potentials). Second, the number of time samples required for ICA to effectively isolate the components is a function of the square of the number of electrodes (Groppe et al., 2009), and studies with large numbers of electrodes may lack the number of time samples needed. Third, the number of ICA components is always equal to the number of electrodes, and some error is introduced by the fact that the number of sources is greater than the number of electrodes (Groppe et al., 2008), although this error may be negligible under many real-word conditions (Mognon et al., 2010). Fourth, although several studies have validated the effectiveness of ICA for blink correction (Frank and Frishkoff, 2007; Hoffmann and Falkenstein, 2008; Mognon et al., 2010), there are fewer validation studies for other types of artifacts. Finally, eye blinks and eye movements do not just create artifactual voltages; they also change the sensory input. For example, the sensory input is massively changed if the eyes are closed when a stimulus appears. Changes in eye position also have a substantial impact on the visibility of a visual stimulus.

For these reasons, it is often necessary to perform artifact rejection rather than, or in addition to, performing artifact correction. Most commercial ERP analysis systems provide only primitive algorithms for identifying trials with artifacts (e.g., rejecting trials on which the overall voltage exceeds a given criterion). ERPLAB contains several different artifact detection algorithms that are tailored to the distinctive properties of the specific artifacts that commonly occur in ERP experiments (as illustrated in **Figure 3**). For example, saccadic eye movements consist of sudden, step-like changes in voltage, and ERPLAB can accurately identify eye movements by computing the cross-covariance between the data and a step function (see Chapter 4 in Luck, 2005). Similarly, amplifier blocking leads to periods of nearly (but not perfectly) constant voltage, and ERPLAB has an algorithm that detects this with high reliability. ERPLAB also contains multiple algorithms for identifying eyeblinks. The user can easily control several parameters to customize the operation of each algorithm. ERPLAB’s artifact detection algorithms have undergone decades of testing and are highly effective.

As in many other ERP analysis packages, ERPLAB’s artifact detection algorithms are applied to segmented EEG data, not continuous EEG data. EEG segments containing artifacts are marked rather than being deleted from the data; marked segments can then be excluded during the process of computing averaged ERP waveforms.

EEGLAB contains its own artifact detection algorithms, including a method for visually inspecting the data and marking segments that contain artifacts (or unmarking segments that were marked by the automatic artifact detection algorithms). These methods can be used instead of, or in combination with, ERPLAB’s artifact detection algorithms.

ERPLAB also has a tool for automatically detecting and deleting segments of the continuous EEG that contain artifacts. The main purpose of this tool is to delete periods of data in which extremely large, idiosyncratic artifacts are present (e.g., when the subject stretches during a break). These artifacts are so large that they may cause ICA to work poorly for ordinary artifacts, and deleting segments of data with these enormous artifacts prior to ICA can improve ICA’s performance.

It is recommended that laboratories establish preset criteria for excluding subjects for whom a large proportion of trials were rejected because of artifacts (see Chap. 4 in Luck, 2005). In our laboratory’s experiments with healthy young adults, for example, we always exclude subjects for whom more than 25% of trials were rejected. This eliminates the possibility that the Type I error rate will be inflated by post hoc exclusion of subjects. ERPLAB therefore provides extensive information about the proportion of trials rejected.

### RE-REFERENCING AND OTHER CHANNEL OPERATIONS

The EEG is typically recorded using differential amplifiers, which provide the difference in potential between two recording electrodes, subtracting out any noise in the ground circuit. Some systems instead record the single-ended voltage between the recording electrode and a ground electrode. In either case, both the recording and reference/ground electrodes contribute equally to the recorded signal.

It is frequently useful to change the reference (or add a reference) offline. For example, if the data are initially recorded using a reference electrode on the left or right mastoid, researchers typically re-reference the data to the average of the left and right mastoids to avoid biasing the data toward one hemisphere (Nunez, 1981; Luck, 2005). Alternatively, it is common to use the average of all scalp sites as the reference, although this requires some caution (Dien, 1998; Luck, 2005). In addition, it is common to re-reference artifact detection electrodes near the eyes into bipolar configurations that maximize the magnitude of ocular artifacts. All major commercial ERP analysis packages have the ability to do this kind of re-referencing, but it is typically implemented either (a) as a “black box” in which it is difficult to know exactly how the re-referencing works or (b) as a large matrix of coefficients that is time-consuming to set up and error prone.

ERPLAB includes a *channel operations* tool that makes re-referencing both easy and transparent. It also makes it easy to perform other channel-related operations, such as interpolation and re-ordering. The essence of this tool is that the end user writes a series of simple equations that describe how the newly created channels should be computed from combination of the existing channels. For example, consider an experiment with 32 channels in which channel 26 corresponds to an electrode over the left eye and channel 27 corresponds to an electrode over the right eye, and the user wants to create a bipolar *vertical electrooculogram* (VEOG) channel by subtracting channel 26 from channel 27. This could be done by writing the following equation:

chan33=chan27−chan26 label VEOG

This creates and adds a channel 33 that is the difference between the existing channels 26 and 27, and gives it the label *VEOG*. This provides a completely transparent and yet simple means of allowing the end user to re-reference the data. Moreover, it provides tremendous flexibility for performing a wide range of operations. For example, one could compute the *norm* of two channels (the square root of the sum of squared channels) and add a 5 μV offset with the following equation:

newchan1=sqrt(chan1ˆ2+chan2ˆ2)+5

To use the average of many electrodes as the reference (e.g., to re-reference to the average of all scalp sites), an *avgchan* function is provided that computes the average of all channels. For example, to re-reference channels 1–3 to the average of channels 1–32 and 38–50, the user would specify the following equations:

$$\begin{array}{c} newchan1=chan1-avgchan\left(1:32,38:50\right)\\ newchan2=chan2-avgchan\left(1:32,38:50\right)\\ newchan3=chan3-avgchan\left(1:32,38:50\right) \end{array}$$

It would be laborious and error-prone for the user to enter individual equations for each of a large number of channels, and a *reference assistant* is therefore included to create the equations automatically. However, the reference assistant does not do the re-referencing directly; it simply creates the equations. The user can then view and edit these equations, making the re-referencing process completely transparent.

It is also simple to write an equation to replace a bad electrode with the average voltage from the surrounding electrodes (i.e., with interpolated values). This can be done by replacing the bad channel rather than by creating a new channel. For example, to replace channel 14 with the average of channels 10, 12, 16, and 18, the user could provide one of the following equations:

chan14=(chan10+chan12+chan16+chan18)/4

or, equivalently,

chan14=avgchan(10,12,16,18)

Operations such as re-referencing and interpolation are most often applied to the EEG, but they are sometimes applied to averaged ERPs. ERPLAB’s channel operations procedure can therefore operate on either EEG or ERP structures.

### FILTERING

In the context of ERP research, filters are often poorly understood and applied inappropriately (see Chap. 5 in Luck, 2005; Yeung et al., 2007). Conventional ERP research is mainly concerned with the time domain (i.e., millisecond-by-millisecond changes in voltage). However, filtering is almost always considered as a frequency-domain operation rather than a time-domain operation. In addition, the most commonly cited virtue of the ERP technique is its temporal resolution, but filters necessarily reduce the temporal precision of the data. Moreover, filters that are ideal in the frequency domain can distort the onset and offset times of ERPs and create artificial peaks and oscillations in the time-domain data (for examples, see Figure 7 in Yeung et al., 2007; Kappenman and Luck, 2010). It is therefore important to think about filters in the time domain and to design filters that produce minimal temporal distortion.

In many commercial ERP analysis systems, filtering is a “black box” procedure in which the details of the filtering are not easily available to the user. For example, most commercial systems do not show the user the filter’s *impulse response function*, which describes how the filter operates in the time domain. ERPLAB is designed to make filtering more transparent to the user, to provide information about the time-domain properties of the filters, and to give the user control over important but underappreciated options.

ERPLAB uses non-causal finite impulse response (FIR) and infinite impulse response (IIR, Butterworth) filters, implemented by means of the Signal Processing Toolbox’s filtfilt() routine. **Figure 2** shows ERPLAB’s filtering GUI. By default, the GUI shows the filter’s frequency response function (**Figure 2A**), but the user can instead view the filter’s impulse response function (**Figure 2B**). The oscillations in the frequency response function shown in **Figure 2B** should be a warning to the user that the specified filter settings may induce artificial peaks or oscillations in the data.

The user can apply a high-pass filter, a low-pass filter, or both (i.e., a bandpass filter). The user specifies the roll-off of the filter (the slope of the filter at its steepest point) and the half-amplitude cutoff of the filter (the frequency at which the amplitude is attenuated by 50%, which is equal to a 6 dB attenuation). When the user specifies the half-amplitude cutoff, the GUI indicates the corresponding half-power value (the frequency at which the power is attenuated by half, which is equal to a 3 dB attenuation). Many ERP researchers appear to be unaware that these values are different and that it is not sufficient to indicate that a filter had, for example, “a cutoff at 30 Hz” without specifying whether this is the half-amplitude or half-power cutoff. By providing both values in the GUI, ERPLAB makes it explicit that these are different values and allows the user to decide which value to report when writing a journal article. This is another example of how ERPLAB implicitly serves an educational purpose.

Filters may produce extremely large distortions at the beginning and end of the waveform being filtered (*edge artifacts*). In ERP research, this problem arises mainly with high-pass filters. ERPLAB uses the standard MATLAB *filtfilt* function for filtering, which includes an algorithm for reducing these edge effects. However, this may not be sufficient to eliminate edge effects in some cases. Consider, for example, a case in which an EEG recording contained a 200 μV voltage offset (which is entirely plausible with DC recordings). The beginning and end of the data epoch would contain implicit transitions from zero to 200 μV, which could potentially lead to large artifacts. To minimize this problem, ERPLAB includes an option for subtracting the mean value of the waveform (the DC offset) prior to filtering.

**FIGURE 4:**
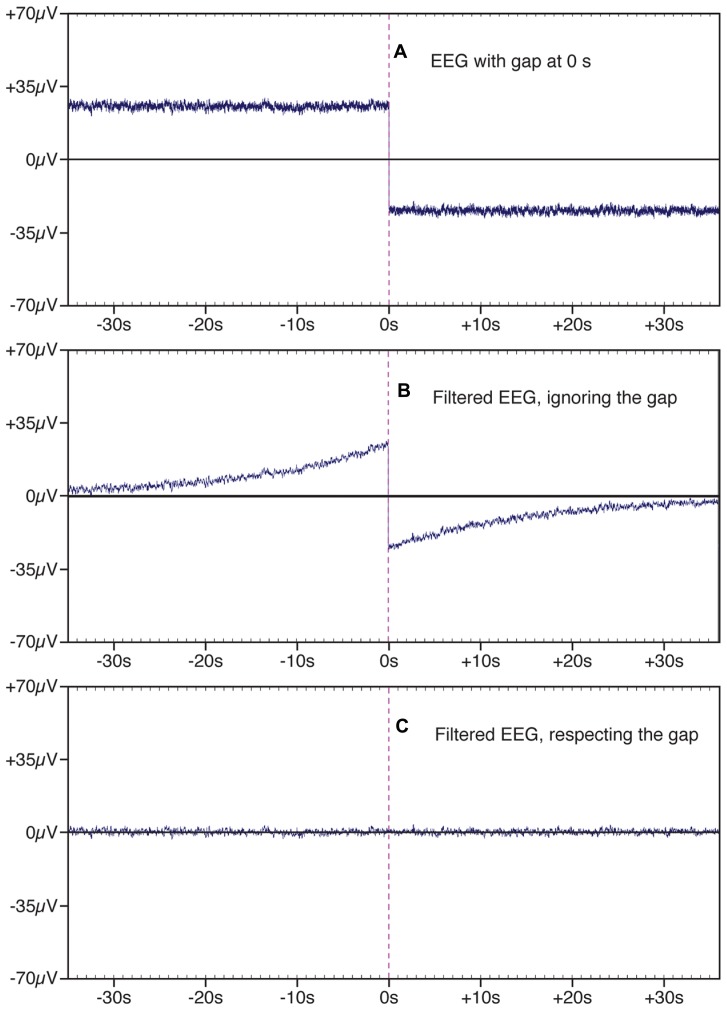
**Example of a segment of EEG data in which there is a gap of a few minutes between the end of one trial block and the beginning of another (shown at time zero).** This gap leads to the appearance of a large and sudden change in the EEG signal at the transition between blocks **(A)**. When this signal was filtered with a bandpass of 0.01–2.0 Hz and a slope of 12 dB/octave **(B)**, the sudden change in the EEG signal at the boundary between blocks led to an artifact that extended forward and backward in time, adding substantial variance to the data. This filter artifact was eliminated by filtering the two trial blocks separately **(C)**.

A related problem arises when a data set includes several blocks of trials, each separated by a gap of a few minutes. The DC offset in the data may change during the gap, leading to a large and sudden shift in the waveform at the transition between trial blocks. **Figure 4A** shows an example of simulated data with a sudden shift produced by a gap between trial blocks, and **Figure 4B** shows the result of filtering the data, using a half-amplitude bandpass of 0.01–20 Hz and a slope of 12 dB/octave. A very large artifact can be seen in the data that extends for almost 30 sec on each side of the gap. To avoid this problem, ERPLAB provides an option for filtering each trial block separately. **Figure 4C** shows that this option eliminates the artifacts at the boundary between trial blocks.

### AVERAGING

The averaging process is relatively simple. All EEG segments that have been assigned to a given bin within a dataset are simply averaged together. The resulting ERPset contains the averaged ERPs for each bin. In some commercial systems, the data from each bin is stored in a separate file, which can lead to a very large number of files in a complex experiment and which makes further processing very tedious. In ERPLAB, all bins are stored together in a single file, so the number of files per subject is small and the application of further processing steps is more efficient.

It is often convenient to store the EEG data from different trial blocks in different files. ERPLAB makes it possible to average across multiple EEG files in a single step.

During averaging, ERPLAB gives the user three options for dealing with EEG segments that have been marked for rejection during the artifact detection process. Specifically, the user may choose to (a) exclude segments marked for rejection, (b) ignore the marks and average all segments, or (c) include only the segments marked for rejection. The last of these options is useful for determining whether the artifacts are consistent and for seeing how they would impact the data if they escaped rejection.

ERPLAB also makes it possible to select random or non-random subsets of EEG epochs for averaging. For example, to compute the split-half reliability of an ERP component measurement, one could average the odd-numbered trials separately from the even-numbered trials and compute the correlation between the component measures from the resulting averaged ERPs. Similarly, it is sometimes useful to equate the number of trials contributing to the averaged waveforms in different conditions, and ERPLAB allows the user to select a random subset of the trials in a given condition for inclusion in the averaged ERP waveforms.

ERPLAB can export the averaged ERP waveforms into a text file using a common file format, allowing the data to be imported into other ERP analysis packages. ERPLAB can also import text files in this format, allowing the user to export data from another package and import it into ERPLAB.

### PLOTTING

ERPLAB toolbox provides simple tools for plotting ERP waveforms and scalp maps. For ERP waveforms, the user has full control of the bins that are overlaid, the channels that are plotted, the time and voltage axes, the font used for labels, etc. For scalp maps, the user can plot 2D or 3D images, can plot maps of individual time points or mean voltages over specified time windows, and can make movies showing changes in topography over time. However, ERPLAB is not designed to directly produce publication-quality figures. Instead, ERPLAB allows users to save the plots as files in several different formats (including portable document format, PDF), which can be imported into any general-purpose graphics program (e.g., Adobe Illustrator).

### DIFFERENCE WAVES AND OTHER BIN OPERATIONS

ERPLAB provides a *bin operations* tool that is used to create difference waves and to perform related operations that involve mathematical recombinations of bins. The bin operations tool is analogous to the channel operations tool but operates on bins rather than on channels (and therefore applies only to averaged ERP waveforms and not to the raw EEG). In an oddball experiment, for example, bin 1 could be used for the standards and bin 2 could be used for the oddballs, and the user would write the following equation to compute an oddball-minus-standard difference wave:

bin3=bin2−bin1 label Oddball−Minus−Stan⁡dard Difference wave

As with channel operations, an enormous number of possible equations can be specified by the user. For example, to compute a waveform that is equivalent to the absolute value of the sum of bins 1 to 4 at each time point, the user could specify the following equation:

bin5=abs(bin1+bin2+bin3+bin4)

In some experiments, it is desirable to combine bins in different ways for different electrode sites. For example, studies of the N2pc component (Luck, 2012a) or the lateralized readiness potential (LRP; Smulders and Miller, 2012) often require the experimenter to compute contralateral-minus-ipsilateral difference waves, in which different channels are contralateral or ipsilateral depending on whether the stimulus or response is on the left or right side. The bin operations tool makes this possible by allowing the user to define electrode groups and then specify which groups should be used in a given part of the equation (e.g., *bin1@LeftElectrodes* + *bin2@RightElectrodes* could be used to combine the left hemisphere electrodes for bin1 with the right hemisphere electrodes for bin2).

### MEASURING AMPLITUDES AND LATENCIES

In the earliest days of ERP research, before general-purpose computers were widely available, specialized hardware was used to record the data, and the output was a set of waveforms plotted on paper. The only easy way to summarize the data from a given subject was to use a ruler to measure the amplitudes and latencies of the peaks in the waveforms (Donchin and Heffley, 1978). The use of peaks to represent the magnitude and timing of the underlying ERP components persisted long after computers took over the job of quantifying the data, even though the peaks often misrepresent the underlying components (see Chapter 2 in Luck, 2005). Over time, however, other approaches have become progressively more common. An important feature of ERPLAB is that it implements several of the approaches that have proven most robust.

**FIGURE 5:**
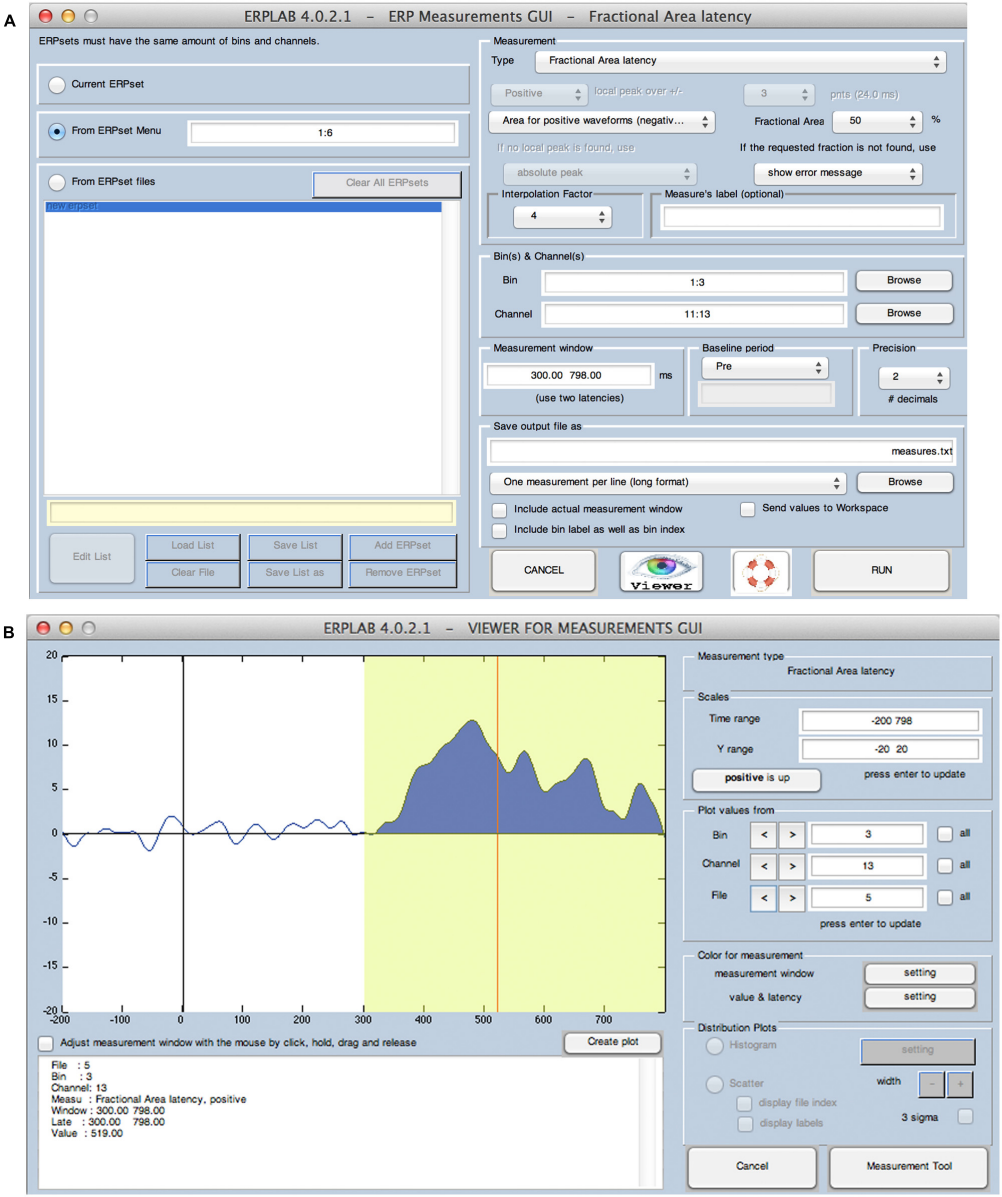
**ERPLAB’s interface for controlling the algorithms used for measuring amplitudes and latencies (A) and for viewing the application of these algorithms to individual ERP waveforms (B)**. In this example, the algorithm measures the area of the positive region between 300 and 798 ms and finds the latency of the point that divides this area into two regions of equal area (the 50% fractional area latency).

Quantification of amplitudes and latencies is achieved in ERPLAB with the *measurement tool*, which is shown in **Figure 5**. The main GUI for this tool (**Figure 5A**) allows the user to specify the ERPsets that will be measured, the measurement algorithm that should be used (e.g., mean amplitude, peak latency), a variety of measurement parameters (e.g., the latency window, the bins and channels to be measured), and the name of a text file that will be used to store the measurements. The contents of the text file can be organized in a manner that is convenient for statistical packages (e.g., one row for each subject, with data from multiple combinations of bins and channels) or in a manner that is more human-readable and is convenient for Excel pivot tables (e.g., one row per measured value, which columns indicating the bins and channel being measured).

Given that single-subject ERP waveforms may differ widely across individuals, it is important to verify that the measurement is working in the desired manner for each subject. The measurement tool therefore includes a *viewer* window (**Figure 5B**), which allows the user to see the measured values superimposed on each waveform being measured.

Several measurement algorithms are available. Peak amplitude and peak latency can be measured, including a *local peak* option that prevents the rising edge of an adjacent component at the edge of the measure window from being chosen as the peak (see Chapter 6 in Luck, 2005). Amplitudes can also be quantified as the mean or area amplitude within a specified time window. When area is measured, the algorithm can find the area of the negative region, the area of the positive region, the sum of the positive and negative areas, or the integral.

**FIGURE 6:**
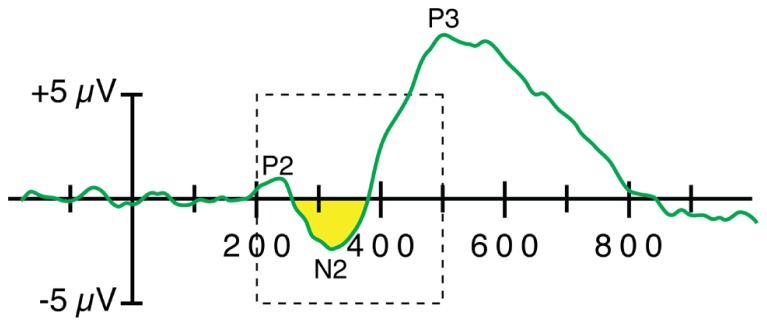
**Application of a *negative area* algorithm to quantify the amplitude of the N2 wave in a single-subject oddball-minus-frequent difference wave.** In this example, the algorithm finds the area of the region that falls below the baseline within a window of 200–500 ms (indicated by the dashed rectangle). The specific time window has very little impact on the measured value, and this algorithm minimizes cancelation of the negative voltage of the N2 wave by the positive voltage of the P2 and P3 waves.

Limiting the algorithm to just the negative region or just the positive region can allow the user to specify a relatively broad measurement window without having negative and positive effects cancel each other. Consider, for example, the oddball-minus-standard difference wave from a single subject that is shown in **Figure 6**. If the goal is to measure the area or mean amplitude of the N2 component, the opposite-polarity P2 and P3 component may partially cancel the N2 component if the measurement window is too wide. A narrow window could be used to avoid this, but N2 latency often varies too much across subjects to define a narrow measurement window that adequately captures the N2 for all subjects. By measuring the area of the negative region (the region that falls below the 0 μV baseline), it is possible to use a broad measurement window without encountering this cancelation. This approach is particularly valuable when the appropriate time window is not known prior to the experiment, especially when it is inappropriate to use the observed waveforms to determine the optimal time window (see e.g., Sawaki et al., 2012).

ERPLAB also implements two approaches to latency measurement that have been demonstrated to be both highly accurate and highly reliable (as shown in rigorous simulation studies by Kiesel et al., 2008). One of these, called the *fractional area latency* algorithm, measures the area within a time window and then finds the time that divides that area into a specified fraction. For example, **Figure 5** shows the use of this algorithm with a fraction of 50%, which means that the algorithm finds the time point that divides the area under the curve into two regions of equal area. A fraction of 20% could be used to find the time at which 20% of the area falls to the left and 80% falls to the right, which can be used to quantify the onset latency of an effect (especially when the measurements are obtained from difference waves). When used with a fraction of 50%, the latency values are highly reliable and can be easily related to median reaction times (see e.g., Luck and Hillyard, 1990; Luck, 1998).

A second algorithm, called *fractional peak latency*, is particularly useful for quantifying the onset of an effect (especially when applied to difference waves). This algorithm finds a peak and then works backward in time until the voltage reaches some fraction of the peak voltage. For example, to estimate the onset latency of the P3 wave in an oddball-minus-standard difference wave, the peak of the P3 would be found and the algorithm would then find the time point at which the voltage reached 50% of this peak (see e.g., Luck et al., 2009). Although it might seem that the 50% point would be consistently later than the actual onset time, this measure is both accurate and robust (Kiesel et al., 2008). One reason is that, because of trial-by-trial latency variability, the onset time of an averaged waveform will be substantially earlier than the average onset of the single trials, and the 50% peak amplitude point may be more representative of the average single-trial onset time (see Chapter 9 in Luck, in press).

These sophisticated methods for quantifying latencies can be limited by the sampling rate of the data. For example, there may not be a time point at which the voltage is exactly 50% of the peak. The measurement tool therefore allows the user to specify an interpolation factor, which is used to increase the precision of the latency measures by applying a spline interpolation to the waveform prior to measurement. This is particularly useful when the *jackknife approach* is used for statistical analysis; as described in the next section, this approach reduces other sources of measurement error so much that it is worthwhile to reduce inaccuracies that can be introduced by discrete temporal sampling.

### STATISTICAL ANALYSES

ERPLAB toolbox does not directly include any statistical functions, but it contains several features designed to facilitate statistical analysis. First, as described in the previous section, the output of the measurement tool can be formatted for major statistical packages, such as SPSS. Second, permutation-based approaches are becoming very popular in ERP research (Blair and Karniski, 1993; Maris and Oostenveld, 2007; Maris, 2012), and ERPLAB contains a *permutation tool* that makes it easy for users to permute the data in various ways. Third, averaged ERPs created in ERPLAB can be read by the *Mass Univariate Toolbox* (Groppe et al., 2011a,b), which provides a means of conducting point-by-point t tests across a large set of time points and channels while controlling the Type I error rate with a variety of sophisticated approaches.

Finally, ERPLAB makes it easy for users to use the jackknife approach, in which the measurements are taken from a series of *leave-one-out* grand averages (grand averages that leave out one subject’s data). The jackknife approach can provide an enormous increase in statistical power for measures that involve non-linear transformation of the data (e.g., onset latency) while controlling the Type I error rate (Miller et al., 1998; Ulrich and Miller, 2001; Kiesel et al., 2008). To facilitate this approach, ERPLAB’s grand averaging tool contains an option for creating *N* leave-one-out grand averages, in which each grand average leaves out one of the *N* subjects. It is then simple for the user to measure amplitudes or latencies from these waveforms, import the results into a statistics package, compute the relevant *t*, *F*, or *r* values, and then perform the jackknife adjustment.

### DOCUMENTATION AND SUPPORT

Extensive documentation for ERPLAB is available at http://erpinfo.org/erplab. This includes an *ERPLAB User’s Manual* that describes every ERPLAB feature in detail. The GUI for each major ERPLAB tool contains a *help* icon that, when clicked, shows the relevant page from this manual. The documentation also includes an *ERPLAB Tutorial* that demonstrates the analysis of a simple experiment in a step-by-step manner. Many of these steps are also demonstrated in a set of *ERPLAB Video Demonstrations*. An *ERPLAB Scripting Guide* is also provided so that users – even those with no prior programming experience – can quickly learn how to go from a series of GUI operations to a script that processes the data from all subjects in an experiment. Finally, a *Frequently Asked Questions* page is available to address issues that commonly arise.

Users can also receive support via email. A general email list provides a forum for posing questions to the entire ERPLAB user community. Users can sign up for this list at http://erpinfo.org/erplab/erplab-email-list. In addition, users can contact the ERPLAB development team directly by email (erplabtoolbox@gmail.com).

## ACCURACY AND BUGS

### ACCURACY

It is important to ensure that a software package produces accurate results. ERPLAB’s tools for computing, transforming, plotting, and measuring waveforms have been extensively tested using both real and simulated EEG/ERP datasets. Using these datasets, we (and our beta testers) compared ERPLAB’s output against the output of three well-known commercial EEG packages: Neuroscan^2^, brain electrical source analysis (BESA^3^), and BrainVision Analyzer^4^. This allowed us to validate the accuracy of ERPLAB’s main processing routines.

### BUGS

Bugs are an inevitable part of any complex software development project. Users are encouraged to report ERPLAB bugs via email to the ERPLAB developers (erplabtoolbox@gmail.com). The ERPLAB development team uses the Trac software system^5^ to track all bug reports and ensure that all bugs are fixed. All significant bugs that have been fixed are reported in the release notes for each new version of ERPLAB. In addition, announcements are sent to the ERPLAB email list when major problems are detected. Moreover, we plan to add a Bug Report List to provide an easy-to-search list of the significant bugs that have been reported for each ERPLAB version.

## CONCLUDING COMMENTS

ERPLAB toolbox provides an inexpensive, easy-to-use, flexible, transparent, and powerful system for analyzing both simple and complex ERP experiments, and it also promotes the understanding and appropriate use of ERP methods. ERPLAB’s GUI dramatically reduces the time required for experienced and novice researchers to learn the package and also aids researchers in learning to write custom scripts. Therefore, ERPLAB has become an excellent alternative to commercial ERP analysis packages. At the time of this writing, it has been publicly available for three years, and the latest version is 4.0. It is stable and reliable, has been downloaded over 7000 times, and has been used in many published papers that examine a broad variety of topics (e.g., Lawhern et al., 2012; Nemrodov and Itier, 2012; Sawaki et al., 2012; Beckes et al., 2013; Leonard et al., 2013; Selzler et al., 2013; Strauss et al., 2013).

## Conflict of Interest Statement

The authors declare that the research was conducted in the absence of any commercial or financial relationships that could be construed as a potential conflict of interest.

## Footnotes

1 http://www.vislab.ucl.ac.uk/

2 http://www.neuroscan.com

3 http://www.besa.de

4 http://www.brainproducts.com

5 http://trac.edgewall.org
